# Association of Sleep-Disordered Breathing and Developing Malocclusion in Children: A Cross-Sectional Study

**DOI:** 10.7759/cureus.39813

**Published:** 2023-06-01

**Authors:** Shweta R Shirke, Amar N Katre

**Affiliations:** 1 Pediatric and Preventive Dentistry, Yerala Medical Trust (YMT) Dental College and Hospital, Navi Mumbai, IND

**Keywords:** angle class of malocclusion, sleep disordered breathing, malocclusion, orthodontic treatment need, children

## Abstract

Background

Sleep Disordered Breathing (SDB) in children and its role in health has received renewed interest in the recent past. Malocclusion is one of the most common multifactorial craniofacial disturbances widely prevalent in children. The primary objective of this study was to assess the association of SDB with developing malocclusion in six to 12-year-old children and the effect of modifiers like age, gender, and tonsillar enlargement.

Materials and method

One hundred and seventy-seven children aged six to 12 years were assessed for developing malocclusion using Angle classification and Index of Orthodontic Treatment Needs (IOTN) comprising of 5 grades. Their parents were administered a pre-validated Pediatric Sleep Questionnaire (PSQ) for assessing SDB, by a single, calibrated examiner. The primary outcomes were SDB score, Angle class of malocclusion, and IOTN grade, assessed as categorical variables. The modifying variables assessed were age, gender, and tonsillar enlargement (Brodsky’s criteria). The data were subject to statistical analysis using Fischer’s test and the odds ratio (OR) was estimated. The modifiers were assessed using logistic regression.

Results

The prevalence of SDB was 69%. SDB has significantly associated with angle class II and class III malocclusion (*x^2 ^*= 9.475, p < 0.05 OR=3.79) and with higher IOTN grades (*x^2 ^*= 109.799, p < 0.05, OR=53.64). Logistic regression revealed that gender and tonsillar enlargement had a significant modifying effect (p<0.05).

Conclusion

SDB had a significant association with developing malocclusion, the odds being higher in angle class II and III malocclusions and higher IOTN grades.

Clinical relevance

Both SDB and developing malocclusion are quite commonly seen in children, though the relation between the two has not been adequately explored. This study shows that they are strongly associated with each other and one could act as a marker for the other.

## Introduction

Sleep Disordered Breathing (SDB) is defined as a disorder of breathing during sleep characterized by snoring, increased upper airway resistance, prolonged and repetitive partial upper airway obstruction, and/ or intermittent complete obstruction essentially disrupting normal ventilation, oxygenation, and sleep quality [1]. SDB ranges from partial to complete upper airway obstruction (obstructive sleep apnea) and can occur throughout childhood from infancy to adolescence [2].

The etiology of SDB is multifactorial and occurs when the balance between factors maintaining airway patency and those promoting airway collapse is disturbed. In children, risk factors include obesity [3], adeno-tonsillar hypertrophy, neuromuscular disorders, and craniofacial anomalies [2].

Children with SDB may exhibit snoring, witnessed apneas, frequent arousals, failure to thrive, nasal congestion, hyperextended neck, recurrent otitis media/ upper respiratory infections (URI), nightmares, daytime sleepiness, restless sleep, enuresis, hyperactivity, drooling, morning headache, insomnia, learning difficulties, delayed puberty, malocclusion (class II or III), overcrowding of teeth, crossbite, mouth breathing and mood disturbances like depression, which are amenable to change with age [4].

As reviewed by Guilleminault, pathophysiological causes leading to abnormal orofacial features such as retrognathia, unilateral or bilateral crossbite, open bite or deep overbite, increased overjet, narrow upper arch, steep mandibular plane, deep hard palate, a long oval face, etc. are commonly seen in children with SDB [5].

Crossbite and open bite malocclusions and the effects of increased upper airway resistance on the dental arch morphology were shown to be associated with SDB in Brazilian and Finnish children respectively [6,7]. Indian adults with SDB had a dental arch abnormality with 60% of the test subjects having an angle class II malocclusion [8]. In a previous pilot study by the same authors, the prevalence of SDB in Indian children was reported to be 48.57% [9].

The dentist is well positioned to screen patients at risk for a sleep disorder and, when adequately trained, contribute to their correction [10]. Though polysomnography is considered the gold standard for confirmatory diagnosis of SDB, screening can be performed with very basic and simple questionnaires. The pediatric sleep questionnaire (PSQ), developed by Chervin et al. (2000) [11] investigated the presence of childhood sleep-related breathing disorders and prominent symptom complexes, including snoring, daytime sleepiness, and related behavioral disturbances. The validity and reliability of the PSQ scale for childhood SDB have been established by comparison with polysomnography-defined obstructive SDB and by the demonstration that the questionnaire scale had the substantial ability to predict diagnostic classification [11]. The PSQ could be used to identify variables that coexist with SDBs in a population for which screening by polysomnography would be impractical [11].

However, there is very limited data on SDB and its relation with developing malocclusion in children, hence, the primary objective of this study was to assess the association of SDB with developing malocclusion in six to 12-year-old children and the effect of modifiers like age, gender, and tonsillar enlargement. Considering that SDB is associated with structural changes in the craniofacial region [6,8], we hypothesize that SDB could be significantly associated with developing malocclusion.

The preliminary results of this research were previously presented as a poster at the International Association of Pediatric Dentistry (IAPD) 2020 Virtual Congress of the International Association of Pediatric Dentistry Global Summit on September 14, 2020.

## Materials and methods

The present study has been reported in accordance with the Strengthening the Reporting of Observational Studies in Epidemiology (STROBE) checklist. An observational cross-sectional study design was used for this study.

Settings and participants

Ethical clearance was obtained from the Institutional Review Board and Ethics Committee of Yerala Medical Trust (YMT) Dental College and Hospital, Kharghar, Navi Mumbai, India (Approval number: YMTDC/IEC/2017/10-12) and written consent was obtained from the parents of children prior to the beginning the study. The protocol was registered at the Clinical Trial Registry India (CTRI) (http://www.ctri.nic.in, ID: CTRI/2018/12/016759).

Six to twelve-year-old children attending the outpatient department (OPD) of the Department of Pediatric and Preventive Dentistry from January 2019 to May 2019 were screened. Children with mixed dentition were included in the study.

Children undergoing or who had completed orthodontic treatment, having ectopically erupting or partially erupted permanent molars (that would interfere with assessment of angle class of malocclusion), with developmental syndromes like cleft lip and/or palate, etc., in whom tonsillectomy/adenoidectomy had been performed, and presenting neurological, neuromuscular or motor disturbances that would hinder with data collection were excluded from the study.

Variables and measurements

The primary variables assessed were SDB [11] and developing malocclusion (using angle class of malocclusion [12] and IOTN [13]; the modifying variables assessed were age, gender, and tonsillar enlargement (using Brodsky’s criteria) [14].

All the children underwent a complete general and oral examination by the same operator. The permanent molar relationship was recorded as angle class I, class II, and class III and its respective subdivisions [12]. The children with an asymmetric class I/ class II molar relationship were recorded under class II malocclusions. Children with an asymmetric class I/ class III molar relationship were recorded under class III malocclusions. Children with an end-on molar relation on one side and class I/ class II/ class III on the other were recorded under class I/ II/ III malocclusions respectively.

SDB was assessed using the 22-point PSQ that assessed parameters such as snoring, daytime sleepiness, daytime behavior problems, enuresis, and hyperactivity under three responses - yes/ no/ don’t know. Parents were asked to tick the most appropriate answer. More than eight positive responses were suggestive of SDB [11].

Impressions were made in alginate hydrocolloid and study models were prepared using dental stone type IV. The IOTN index, comprising five grades, based on treatment need with grade 1 indicative of no treatment need and grade 5 indicative of very great treatment need [13], was assessed using clinical parameters and the stone models.

The operator was calibrated for assessment of the angle class of malocclusion, grade of tonsillar enlargement, palatal depth measurement, and IOTN, two assessments were made at an interval of seven days.

Assuming, from existing literature, a difference in the prevalence of SDB [15] to be 10% in this study population with alpha at 0.05 and beta < 0.2 (power > 80%), the number of children required for the study was 131. Accounting for 20% attrition due to non-compliance and other factors, the sample size was increased to 161. To account for operator calibration, an additional 10% of children were recruited. Hence, the total sample size was 177.

Statistical analysis

The data were coded and organized using MS Excel (Microsoft Corporation, 2018) and assessed using MedCalc Statistical Software version 13.3.1 (MedCalc Software, Ostend, Belgium). PSQ score and grade of tonsillar enlargement were expressed as categorical variables with percentages. Data for IOTN and Angle’s molar relation were expressed as percentages for each category. The association between PSQ scores, IOTN, and Angle’s molar relation was assessed using Fischer’s test. Data for angle class of malocclusion and IOTN grades were dichotomized (angle class I vs. angle class II and III, IOTN grades 1,2,3 vs. 4,5) and odds ratio (OR) was estimated. Intra-examiner reliability was assessed using Cronbach’s alpha. The statistician was blind to the coding.

## Results

The total sample consisted of 177 children out of which 16 children were considered for intra-examiner reliability. The data from these additional 16 children were not included in the final analysis. The Cronbach’s alpha score for intra-examiner reliability for palatal depth and IOTN was 0.99, 0.73 for the angle class of malocclusion, and 1.0 for tonsillar enlargement.

A hundred and sixty-one participants were recruited in the study, of which one participant was excluded as the molar relation was unclear. Hence, the outcomes of 160 participants were subjected to analysis.

The overall prevalence of children with a risk of SDB using PSQ was 69%, of whom 57.27% were girls. 69% of children had class I molar relation, whereas 26% of children had class II molar relation, amongst them 93% were class II division 1 and 7% were class II division 2. 5% of children had class III molar relation. 43% of children showed grade 4 IOTN (68% - grade 4a, 13% - grade 4b, 19% - grade 4d) i.e., great need of treatment required, and 6% of children showed grade 5 i.e., very great need of treatment. The most common type of tonsillar enlargement was grade +2 (33%) and grade +3 (32%) (Table 1).

**Table 1:** Distribution of the study population

AGE (Years)		Gender		SDB		Molar relation (Angle Class)			Tonsillar enlargement (Brodsky’s criteria)					OTN (Grades)				
(N = 161)		(N = 161)		(n = 160)		(n = 160)			(n = 160)					(n = 160)				
6<9	9<12	M	F	Present	Absent	I	II	III	0	1+	2+	3+	4+	1	2	3	4	5
%	%	%	%	%	%	%	%	%	%	%	%	%	%	%	%	%	%	%
63.3 (n=102)	36.6 (n=59)	46.6 (n=75)	53.4 (n=86)	68.8 (n=110)	31.2 (n=50)	68.8 (n=110)	26.2 (n=42)	5 (n=18)	11.9 (n=19)	18.1 (n=29)	33.1 (n=53)	31.9 (n=51)	5 (n=8)	26.9 (n=43)	10.6 (n=17)	13.8 (n=22)	43.1 (n=69)	5.6 (n=9)

Intergroup comparison for molar relation showed that 85.4% of children with class II molar relation and 87.5% of children with class III molar relation had SDB as against 61.3% with class I molar relation (x2 = 9.475, p < 0.05) (OR=3.79, 1.56-9.20) (Table 2).

**Table 2:** Association of SDB and molar relation (*p < 0.05 - Significant), # - dichotomised as Class I (Ref) vs. Class II and III, N = 160

			SDB		x^2^	p	OR^#^ (CI)
			Absent	Present			
Molar Relationship	CLASS I	n = 111	43	68	9.475	0.009*	3.7941 (1.5633, 9.208)
		within molar relationship	38.7%	61.3%			
		within SDB	86.0%	61.8%			
	CLASS II	n= 41	6	35			
		within molar relationship	14.6%	85.4%			
		within SDB	12.0%	31.8%			
	CLASS III	n = 8	1	7			
		within molar relationship	12.5%	87.5%			
		within SDB	2.0%	6.4%			

Intergroup comparison for IOTN grades showed that 98.6% with grade 4 IOTN had SDB as against 7% with grade 1 IOTN (x2 = 109.799, p < 0.05) (OR=53.64, 12.32-233.59) (Table 3).

**Table 3:** Association of SDB and IOTN (*p < 0.05 - Significant), # - dichotomised as Grade 1, 2,3 (Ref) vs. Grade 4,5, N = 160

			SDB		x^2^	p value	OR^#^ (CI)
			Absent	Present			
IOTN	Grade 1 No treatment required	n = 43 (26.9%)	40	3	109.799	0.000*	
		within IOTN	93.0%	7.0%			53.647 (12.32, 233.59)
		within SDB	80.0%	2.7%			
	Grade 2 Little treatment required	n = 17 (10.6%)	5	12			
		% within IOTN	29.4%	70.6%			
		% within SDB	10.0%	10.9%			
	Grade 3 Borderline	n = 22 (13.8%)	3	19			
		% within IOTN	13.6%	86.4%			
		% within SDB	6.0%	17.3%			
	Grade 4 Great Need of treatment	n = 69 (43.1%)	1	68			
		% within IOTN	1.4%	98.6%			
		% within SDB	2.0%	61.8%			
	Grade 5 Very Great Need of treatment	n = 9 (5.6%)	1	8			
		% within IOTN	11.1%	88.9%			
		% within SDB	2.0%	7.3%			

Logistic regression revealed a significant association of IOTN and molar relation with SDB (p < 0.05) with gender {(B=0.254), (C.I.=0.082-0.788)} and tonsillar enlargement [(B=7.689), (C.I.= 3.632-16.277)] as the modifier variables (Table 4). Age had no significant modifier effect.

**Table 4:** Logistic regression of variables and the effects on association of SDB and developing malocclusion (*p < 0.05 - Significant)

Variables	Exp(β)	95% C.I.	p
Gender	0.254	0.082 - 0.788	0.018*
Tonsillar Enlargement (Brodsky’s Criteria)	7.689	3.632 - 16.277	0.000*

## Discussion

The interruption of normal sleep and breathing patterns leading to reduced oxygen levels has been shown to trigger a cascade of events in children with SDB [16], growth impairment being one. Hypertrophy of the tonsils can lead to difficulty in swallowing and interfere with adequate caloric intake, also causing growth impairment [17]. Mouth breathing has also been found to be a cause of abnormal facial development such as adenoid faces and dental malocclusion [18].

SDB can affect the development of the craniofacial complex which in turn may affect the dental and skeletal relationship [19], thereby emphasizing the need of diagnosing the cause of developing malocclusion. Pediatric dentists can observe and guide the developing dentition and also explore and treat the cause, as much as intercepting the problem; correction of SDB may improve the orthodontic outcomes or vice versa. However, it is essential to assess the prevalence of SDB in children and its relation with developing malocclusion, hence the present observational cross-sectional study is justified.

One hundred and seventy-seven children between six to 12 years of age who visited the Department of Pediatric and Preventive Dentistry for dental treatment procedures were included in the study, of which the data from sixteen children were utilized for operator calibration. Owing to the possible errors as part of this process, especially in assessing molar relations, these data were not included in the final analysis. The American Academy of Orthodontists (AAO) recommends the first orthodontic evaluation as early as six to seven years of age for all children [20]. Researchers have also stated that early myofunctional treatment during mixed dentition contributes to treating malocclusion [21], hence this age group was selected.

Informed consent of the parents of the children recruited for the study was obtained in accordance with the Indian Council of Medical Research (ICMR) guidelines. A pre-validated Pediatric Sleep Questionnaire (PSQ) was used in this study to assess SDB in children. Questionnaires could serve as valid and reliable instruments that can be used to identify SDB or risk of SDB, which can be later, if necessary, confirmed with polysomnography [22]. Though polysomnography is the gold standard for the diagnosis of SDB, the time, effort, and expense of laboratory studies have limited relevant research, particularly epidemiological research that requires large samples. PSQ is a valid and reliable instrument that can be used to identify SDBs or associated symptom constructs in clinical research when polysomnography is not feasible. PSQ can be used over a broad age range and provides a more comprehensive assessment compared to other questionnaires [23].

Angle class of malocclusion was used to assess the molar relation. It is a useful, simple, chairside semantic measure for the assessment of malocclusion; however, treatment cannot be based on it alone. The IOTN index was used to measure the severity of malocclusion and orthodontic treatment needs. It is an objective, categorical yet quantifiable, economical, validated, and non-invasive scale which has been used quite widely [24].

Most children in our study had SDB with most belonging to the age group of six to nine years. This is in accordance with previous studies that SDB is more prevalent in five to nine-year-old children with snoring as the most common symptom [25].

SDB was seen in children with all types of molar relations, notably more prevalent in class II and class III molar relations. The present study indicates that children with sleep-related breathing disorders have differences in dental arch dimensions. We found that increased overjet and a retro-positioned maxillary and/or a mandibular dentition could be associated with lowered tongue position (may/ may not be associated with mouth breathing); lowered tongue position with or without tongue tie is a known risk factor for SDB. A lowered and more posterior tongue position may lead to a reduction in pharyngeal airway space and a resultant decrease in airflow during sleep, pre-disposing the individual to SDB [26]. Therefore, orthodontic interventions like maxillary expansion and forward mandibular positioning, supplemented with tongue-tie surgeries (as suggested by Camacho et al.) may be useful in correcting sleep related breathing disorders.

Most of the children with SDB exhibited grade 4 IOTN followed by grade 5 IOTN, indicative of a great and very great need for orthodontic treatment respectively. Most of the children with grade 4 IOTN had an increased overjet (grade 4a) whereas the remaining exhibited increased overbite, reverse overjet, crowding, and other forms of malocclusion. Increased overjet is usually indicative of a distal molar occlusion and mandibular deficiency, whereas increased overbite usually reflects a reduced intraoral intermaxillary space and a decreased lower anterior facial height [27]. Pirilä et al. has reported that increased overjet was seen most in SDB and snoring subjects [7]. No previous study reports the relation of operator-assessed IOTN with SDB. 

A recent systematic review concluded that no firm conclusion can be drawn regarding an association between specific malocclusion traits and SDB. The review reported no association between molar relationship and crowding and SDB symptoms in children [28]. However, we found that the odds of having SDB were much higher in children with malocclusion (class II and class III and with higher grades of IOTN), suggestive that SDB has a strong association with malocclusion, which was in accordance with our hypothesis; the same can be further substantiated using more robust study designs. Both SDB and malocclusion are an outcome of improper craniofacial development. Angles class II and III molar relations may be indicative of abnormal maxillomandibular developments. Hence it may be plausible that our study found a significant association. None of the studies included in the systematic review assessed IOTN. IOTN was used in this study as it may guide the clinician in making a clinical decision on orthodontic treatment. Overjet, cross bites, and open bites are assessed in IOTN; a significant association with SDB and these parameters are a part of the conclusion of the systematic review. Though the temporality of SDB may be assumed in the causation of malocclusion, this study being cross-sectional in nature, could not prove the cause-effect relation.

Tonsillar enlargement had an influence on the association of SDB and developing malocclusion in our study. This is in accordance with a previous study by Lee et al. suggesting that due to adenoidal enlargement, soft tissues become functionally impaired resulting in continuous mouth breathing and nasal disuse [29].

Gender had a significant effect on the association of SDB and developing malocclusion. SDB and developing malocclusion was seen more often in girls than boys. This is in agreement with previous studies by Bixler et al. wherein girls were more affected by SDB than boys [30].

In the present study, age did not have any modifying effect on the association between SDB and malocclusion.

Being a cross-sectional study, only the association between SDB and developing malocclusion was assessed, however, a causality could not be established. This study assessed IOTN in the developing (mixed) dentition; it could vary in the permanent dentition after considering factors such as dental age variations, growth spurts, self-correcting anomalies, etc. The PSQ, though a validated tool that can be easily self-administered, has inherent limitations, it is a questionnaire, and the effect of recall bias on the responses of the parents when addressing the questionnaire cannot be overlooked. We attempted to overcome the bias by recording the observations after three days. We could assess the presence of SDB; however, the magnitude of the problem was beyond the scope of the questionnaire. Additionally, a diagnostic sleep study with a PSG was not part of this research.

The sample was drawn from children aged six to 12 years visiting the Department of Pediatric and Preventive Dentistry in a metro city seeking treatment. Therefore, the results of this study may be generalizable to children of similar age groups in similar study settings.

## Conclusions

SDB, assessed using PSQ, had a strong association with developing malocclusion, assessed with angle class of malocclusion and IOTN, suggesting that developing malocclusion could serve as a marker for SDB and vice versa. A higher prevalence of SDB was seen to be associated with angles class II and class III malocclusions. SDB was significantly associated with higher grades of IOTN. Gender and tonsillar enlargement had a significant modifier effect on the association between SDB and developing malocclusion. Age did not have any modifying effect.
